# Maternal Vaccine Acceptance and Attitudes Before and After the COVID-19 Pandemic: A Narrative Literature Review

**DOI:** 10.3390/vaccines14060536

**Published:** 2026-06-17

**Authors:** Barbara Frączek, Karolina Pieniawska-Śmiech, Mateusz Babicki, Bartosz Balcer, Natalia Dolata, Dagmara Pokorna-Kałwak, Karolina Kłoda

**Affiliations:** 1 Indywidualna Specjalistyczna Praktyka Lekarska Barbara Frączek, ul. Brzeziny 1/6, 72-005 Warzymice, Poland; barbaramaziakowska@gmail.com; 2 3rd Department and Clinic of Paediatrics, Immunology and Rheumatology of Developmental Age, Wroclaw Medical University, 51-149 Wroclaw, Poland; 3 Department of Immunology and Pediatrics, The J. Gromkowski Provincial Specialist Hospital, 51-149 Wroclaw, Poland; 4 Department of Family Medicine, Faculty of Medicine, Wroclaw Medical University, 50-367 Wroclaw, Poland; ma.babicki@gmail.com (M.B.); daga_kalwak@o2.pl (D.P.-K.); 5 Institute of Heart Diseases, Jan Mikulicz Radecki University Clinical Hospital, 50-556 Wroclaw, Poland; bartosz.balcer10@gmail.com; 6 University Centre of General Dermatology and Oncodermatology, Jan Mikulicz Radecki University Clinical Hospital, 50-556 Wroclaw, Poland; nataliadolata1998@gmail.com; 7 MEDFIT Karolina Kłoda, ul. Narutowicza 13E/11, 70-240 Szczecin, Poland; wikarla@gazeta.pl

**Keywords:** vaccinations against COVID-19, vaccinations against influenza, vaccinations against pertussis, vaccinations against RSV, vaccinations hesitancy in pregnant women, vaccinations in pregnant women

## Abstract

Objectives: This study aims to assess the acceptance of vaccinations among pregnant women, particularly against influenza, pertussis, COVID-19, and RSV, and to identify factors influencing their willingness to get vaccinated. It also seeks to evaluate the impact of the COVID-19 pandemic on maternal attitudes and behaviors regarding vaccination. Methods: The analysis involved a review of existing literature and studies to evaluate the level of vaccine acceptance among pregnant women before and after the COVID-19 pandemic. Factors contributing to vaccine hesitancy, including misinformation, lack of knowledge, and the influence of healthcare professionals, were examined. Results: The findings indicated that, despite scientific evidence supporting the safety and efficacy of vaccines during pregnancy, public concerns remain about their impact on the developing fetus. The outbreak of the COVID-19 pandemic has increased awareness of the risk of infectious diseases, but at the same time, its impact on vaccination rates among pregnant women is ambiguous and geographically diverse. Misinformation and decreased access to healthcare during the pandemic negatively affected vaccine uptake. Trustworthy information provided by healthcare professionals emerged as a key factor in promoting vaccine acceptance. Conclusions: To improve vaccination rates among pregnant women, it is essential to provide clear, evidence-based information through healthcare professionals, particularly those directly caring for pregnant women. Educational campaigns should address concerns calmly and without judgment, emphasizing the safety and benefits of vaccinations. Enhanced access to healthcare and vaccinations, along with strategic information dissemination, can significantly improve vaccine acceptance during pregnancy. Lessons learned from past pandemics should be incorporated into the development of healthcare strategies aimed at implementing recommended vaccinations for pregnant women in the future.

## 1. Introduction

Vaccinations recommended during pregnancy include those against influenza, pertussis, and, in recent years, both COVID-19 and respiratory syncytial virus (RSV) [1,2,3,4,5]. They are intended to protect the health of pregnant women, the developing fetus and the newborn [6,7]. After receiving the vaccine, the mother’s body begins producing antibodies (active immunity), and then the IgG antibodies are transported across the placenta to the fetus, providing passive immunity against specific diseases. This provides temporary protection for the child during a particularly vulnerable period, when the child’s immune system is relatively immature during the first months of life and needs time to receive the first vaccines and develop its own active immunity [8]. Despite numerous scientific studies confirming the effectiveness and safety of vaccinations recommended during pregnancy, their use still raises concerns [5,6,9,10]. Questions arise in relation to the need for vaccine administration and its effectiveness [9,10,11,12]. The main doubts regard their potential impact on the developing fetus [11,12]. However, according to studies, no significant increase in adverse pregnancy or neonatal outcomes was associated with maternal vaccination [13,14]. In recent years, the acceptance of vaccinations by pregnant women in many countries has increased, but often remains at an insufficient level [15,16]. The outbreak of the COVID-19 pandemic, on the one hand, increased interest and the level of awareness about the safety and effectiveness of vaccinations, but at the same time, it limited access to routine medical care and raised doubts related to the introduction of new vaccines [17,18,19]. In most countries, there are no official statistics on the level of vaccination coverage of pregnant women for specific pathogens. The data come mainly from scientific research conducted on specific groups of people, in specific locations, or from online surveys. Therefore, results may vary depending on the population studied.

The aim of this narrative review was to summarize the current evidence on maternal vaccination acceptance against influenza, pertussis, COVID-19, and RSV and to identify factors associated with vaccine uptake, acceptance, hesitancy, and willingness to vaccinate. Particular attention was paid to the potential impact of the COVID-19 pandemic on maternal attitudes and behaviors regarding vaccination.

## 2. Materials and Methods

The review of the literature was based on scientific articles indexed in the PubMed database and performed between January 2019 and June 2026, in order to capture evidence from the period immediately preceding, during, and following the COVID-19 pandemic. However, selected older publications were included when they represented foundational studies, historical policy milestones, or essential background information relevant to maternal immunization. Only English-language articles published during this period were included in the search. To minimize bias, there were no restrictions on publication types, but grey literature and non-peer-reviewed articles were not taken into account. Official governmental or professional guidance documents were used to provide current recommendations, policy context, or regulatory information. These sources were not included in the evidence synthesis itself.

The search strategy combined Medical Subject Headings (MeSH) and free-text terms related to maternal vaccination. It was not tailored to any specific region of the world. The following keywords and phrases, entered one at a time, were used to identify the studies of interest: influenza vaccination, pertussis vaccination, COVID-19 vaccination, RSV vaccination, vaccination of pregnant women, vaccination during pregnancy, vaccination acceptance. Additional relevant publications were identified through manual screening of reference lists from eligible articles. Articles were classified as eligible when they (1) reported original research, systematic reviews, meta-analyses, or large population-based studies; (2) included pregnant women or women planning a pregnancy; (3) examined vaccination uptake, acceptance, willingness to vaccinate, vaccine hesitancy, or factors influencing vaccination decisions; and (4) focused on influenza, pertussis (Tdap), COVID-19, or RSV vaccination. Exclusion criteria were (1) consisted solely of editorials, commentaries, conference abstracts, or non-scientific publications; (2) focused on childhood or general adult vaccination without maternal vaccination data; and (3) non-English articles. It is important to note that the data mainly come from scientific research conducted on specific groups of people, in specific locations, or from online surveys. Therefore, results may vary depending on the population studied. Relevant information was extracted from each publication, including country, study design, study population, sample size, vaccine type, outcome measures, vaccination uptake or acceptance estimates, and factors associated with vaccination decisions.

Given the heterogeneity of study designs, populations, outcome definitions, and measurement methods, a quantitative meta-analysis was not feasible. Therefore, findings were synthesized narratively and organized according to vaccine type (influenza, pertussis, COVID-19, and RSV), with particular attention paid to changes observed before, during, and after the COVID-19 pandemic.

This study did not require the approval of the Bioethics Committee.

## 3. Results

### 3.1. Influenza Vaccination

Influenza poses an underestimated but high risk of morbidity and mortality, especially for individuals over 65, those with chronic conditions, pregnant women, newborns, and infants [20]. Pregnant women are at greater risk of serious complications, such as severe pneumonia, a higher risk of hospitalization, admission to intensive care units, and adverse perinatal and neonatal outcomes such as preterm birth, low birth weight, miscarriage, and fetal death [7,20]. Newborns and infants under 6 months are also at higher risk but cannot be vaccinated. To protect them, maternal vaccination is crucial, as antibodies pass through the placenta to the fetus, providing protection in early life [6]. Additionally, the cocoon strategy—vaccinating parents and close contacts—helps reduce virus transmission to infants [6,20].

The history of flu vaccinations dates back to the 1940s. Influenza pandemics, such as the 1957 outbreak caused by the H2N2 virus and the 1970s outbreak caused by the H1N1 virus, contributed to the introduction of vaccination programs [21]. The World Health Organization (WHO) has been recommending influenza vaccination for all pregnant women, regardless of the stage of pregnancy, since 2012 [22]. This is also confirmed by numerous recommendations around the world [23]. The recommendation of the Advisory Committee on Immunization Practices (ACIP) regarding the vaccination of pregnant women against influenza was introduced in 1995, extended to all trimesters in 2004, and since 2010, it has advised annual vaccination for the entire population over 6 months of age, including pregnant women [3,21]. The Centers for Disease Control and Prevention (CDC) recommends annual influenza vaccination for everyone over 6 months of age, especially those at high risk, such as pregnant women and people with chronic diseases [24]. Similarly, the European Centre for Disease Prevention and Control (ECDC) and the National Health Service (NHS) in England recommend annual influenza vaccination for high-risk groups, including pregnant women, children aged 6 months to 5 years, older people, and those with chronic diseases, and by 2014, 42% of WHO Member States had introduced an official policy of vaccinating pregnant women against influenza [1,22]. The introduction of official recommendations caused a significant increase in vaccination rates in this population. For instance, in Great Britain, the vaccination rate increased by 17% between 2012 and 2020 [3,25]. Similarly, this increase was observed in many other highly developed countries- in Spain and the United States, an increase of approximately 50% was observed after the introduction of these recommendations [3,26,27,28,29,30,31,32]. Despite numerous recommendations from many global organizations, the uptake of influenza vaccinations among pregnant women, both in Europe and worldwide, is still insufficient. These decisions are largely influenced by the education provided by gynecologists and the reliable information they provide about scientifically supported benefits for pregnant women [33]. Research on this topic was carried out, among others, in France, Italy, and China. It has been shown that despite official guidelines, medical staff rarely recommend vaccination against influenza to pregnant women; as a result, the percentage of vaccinated people in these countries is very low, which shows how important the advice from medical professionals is [5,29,34,35,36,37]. Education about the benefits and safety of influenza vaccination for pregnant women is crucial to increasing its acceptance. A significantly higher vaccination rate was observed in countries where information programs are conducted. A study by Carlson et al. evaluated a 2016 public health campaign in New South Wales, Australia. Vaccination uptake in 2016 was around 54%, higher than earlier estimates from previous years, suggesting improvement over time. Many women reported that healthcare providers (especially midwives and doctors) were still the most influential source of information, more than media campaigns. Social media was useful for reach, but its measured behavioural impact appeared limited compared with clinical recommendations [38]. Another research from Australia, which was a randomized controlled trial performed between 2022 and 2024, tested behavioural nudges delivered via SMS messages to pregnant women to improve both influenza and COVID-19 vaccination uptake. The nudges delivered via SMS resulted in small, not statistically significant increases in COVID-19 and influenza vaccination uptake among pregnant women [39]. According to the majority of study participants who completed an online survey, clinician recommendations remain far more influential [39].

Kazakhstan also introduced a successful flu vaccination program for pregnant women. It included an annual educational program for women of reproductive age, providing information through the mass media, but also training for healthcare workers on vaccinations for pregnant women. The results were impressive. The percentage of pregnant women vaccinated against influenza increased from just over 4% in season 2011/2012 to over 90% in season 2015/2016 [37,40]. However, it should be noted that these data are reported by the Kazakh authorities and cited by the WHO as an example of a successful vaccination program. It is worth mentioning that vaccination of pregnant women in Kazakhstan began as a public program in 2012, which explains the very low starting point [37,40]. A notable limitation is that the reported coverage estimates are derived from a WHO implementation report rather than a peer-reviewed primary publication. Consequently, the underlying methodology and validation procedures are not described in sufficient detail to allow independent assessment. Similarly, in South Korea, the percentage of women vaccinated against influenza during pregnancy increased from 4% to almost 60% over the period 2006–2019. This was due to a higher frequency of vaccination advice by doctors and broader knowledge, awareness, and perception of vaccinations [28,41]. According to studies, health professionals play a crucial role in the decision-making process as they are the most trusted sources of health- and vaccine-related information for pregnant women [9,41]. Therefore, education for obstetricians, midwives, community midwives, and nurses about vaccinations has a particularly preventative role and should incorporate reliable data on their safety and effectiveness.

Access to vaccines is another factor that plays an important role. In some countries where influenza vaccinations are free and widely available, vaccination rates may be higher. For instance, New Zealand has introduced a free vaccination program for pregnant women at primary care physicians, obstetricians, midwives, and pharmacies. Over the years 2013–2018, an over 20% increase in the number of pregnant women vaccinated against influenza was demonstrated [42]. This is also confirmed by observations from other studies, which clearly indicate that the willingness to get vaccinated increases significantly when it is free [43].

Vaccination against influenza among pregnant women varies significantly depending on the region of the world. Significantly more pregnant women are vaccinated against influenza in highly developed countries than in those with lower average per capita income. This is largely related to the official vaccination policy. Based on average per capita income, 50% of countries recommending influenza vaccination in pregnancy are high-middle-income countries [22].

Official recommendations to vaccinate pregnant women against influenza have been introduced, for example, in India, Lima, and Bangkok. Despite this, only 8% of pregnant women in India are aware of the risk of influenza. In Lima and Bangkok, over 90% of pregnant women know about this disease. However, the number of pregnant women vaccinated against flu in these cities varies significantly. In Lima, this is half of the surveyed women, while in Bangkok, it is only 3%. The research conclusions indicate that the key factor influencing the willingness to get vaccinated against influenza during pregnancy is informing and recommending vaccinations by doctors. It has been shown that only 1% of doctors in India recommend influenza vaccination for pregnant women. In Lima, it is about 60%, and in Bangkok, it is 17%. Prenatal visits are an opportunity to recommend vaccination. Lack of recommendations from healthcare professionals and lack of access to medical services are the main factors contributing to the low vaccination rate in these areas [29]. There are significant differences in knowledge, attitudes, and practices related to influenza and influenza vaccination across countries in South and Southeast Asia, South America, and Africa. Many low- and middle-income countries have not implemented official flu vaccination policies yet. Monitoring programs, if they already exist, mainly focus on tetanus in newborns. In a study in Kenya, almost a third of women had never heard of the flu. Among women who were aware of such a disease, over 80% would be willing to take the vaccine if they had the opportunity [44,45]. In Afghanistan, despite WHO recommendations in 2021, it was found that almost 90% of surveyed women had never heard of influenza vaccination, but over 70% were willing to take the vaccine after gaining knowledge about it [46].

The impact of the COVID-19 pandemic on influenza vaccination uptake among pregnant women is inconclusive. A prospective cohort study conducted at the National Maternity Hospital in Dublin in 2022 reported that 57% of pregnant women were vaccinated against seasonal influenza that year, which was a significant increase from 39% in a similar study in 2016 [47]. A study conducted in China during the COVID-19 pandemic reported an overall acceptance rate of 76.5% (95% CI: 74.8–78.1%) among 2568 registered pregnant women. However, concerns about vaccine safety remained a significant barrier [48]. By comparison, a cross-sectional study using an online questionnaire conducted in Korea found that 51% of women participating in the study were vaccinated against influenza. Factors associated with influenza vaccine acceptance included knowledge about the influenza vaccine, trust in healthcare professionals, and COVID-19 vaccination during pregnancy. The COVID-19 pandemic did not affect the influenza vaccination rate, nor did it affect the acceptance of the influenza vaccine among the majority of pregnant women in Korea [49]. A fully online survey and self-assessment study conducted in Poland during the pandemic showed that 52% of surveyed women responded that influenza vaccination during pregnancy is safe. However, only 21% of women had received the influenza vaccine during their current pregnancy, and 17.5% planned to get vaccinated [19]. The most interesting changes were observed in the United States. In a study conducted by Irving et al., influenza vaccination coverage among pregnant women increased from 63.0% during the 2016–2017 season to a peak of 71.0% in the 2019–2020 season. Following the onset of the COVID-19 pandemic, vaccination uptake declined, reaching 56.4% in the 2021–2022 season. Across all six influenza seasons analyzed, the lowest vaccination rates were consistently observed among pregnant women aged 18–24 years and among non-Hispanic Black women. Notably, the 2021–2022 season was associated with the lowest vaccination coverage across all age, racial, and ethnic groups. The authors suggested that the decline in influenza vaccine uptake may have been influenced by the spread of misinformation, increasing vaccine hesitancy and distrust, as well as reduced access to healthcare services during the COVID-19 pandemic [50].

To sum up, the impact of the COVID-19 pandemic on influenza vaccination coverage among pregnant women has been heterogeneous; while some countries have reported increased vaccination uptake and acceptance, others have observed a decline. The level of influenza vaccination among pregnant women is still insufficient. Periodic declines in influenza vaccination coverage among pregnant women in some regions indicate the need for further efforts to improve coverage among pregnant women. The willingness to accept the vaccine is positively influenced by the introduction of recommendations from scientific societies and the introduction of information programs. Significantly more women are willing to get vaccinated after receiving such a recommendation from a doctor. On the other hand, women uninsured, with a lower level of education, less access to medical care, and less frequent visits during pregnancy are much less willing to undergo this vaccination. Factors contributing to the low acceptance of influenza vaccination also include erroneous beliefs about the risk of influenza infection for oneself or the fetus, doubts about the safety and effectiveness of the vaccine, and the negative influence of the media. Knowledge about the safety and benefits of influenza vaccination among healthcare workers is also insufficient—few have been vaccinated and recommend vaccination, especially to pregnant women. Recommendations from doctors remain crucial for a woman’s decision to undergo influenza vaccination during pregnancy [9,35,51,52,53].

Multiple systematic reviews and large observational cohort studies have found no evidence that inactivated influenza vaccination during pregnancy increases the risk of spontaneous abortion, stillbirth, congenital anomalies, preterm birth, small-for-gestational-age birth, or major maternal adverse events [54].

### 3.2. Vaccination Against Pertussis

Pertussis is a respiratory disease particularly dangerous for young children, potentially leading to severe pneumonia, brain damage, and even death, especially in those under six months of age. Vaccination is available from six weeks of age, but newborns and younger infants remain at risk [55]. Vaccinating pregnant women is recommended to protect their babies, as antibodies produced in vaccinated mothers pass through the placenta, offering protection during the first months of life [6]. Due to the short survival period of antibodies, the Tdap vaccine is advised at 27–36 weeks of pregnancy during each pregnancy, a recommendation also supported by countries such as the United Kingdom, New Zealand, Australia, and many others [2,3,56]. The World Health Organization has recommended pertussis vaccination for every pregnant woman since 2015 [3]. Additionally, vaccinating mothers reduces their risk of infection and transmission to their babies, and vaccinating family members, following the cocooning strategy, helps protect young children [2,6,56]. Importantly, countries that have implemented routine maternal pertussis vaccination programs have reported substantial declines in national rates of pertussis among young infants, underscoring the effectiveness of this preventive strategy at the population level [55].

Since 2023, there has been an increase in pertussis cases worldwide, making the resurgence of this disease a global public health challenge for some time [50]. Data from the National Health Service in England shows a rapid increase in pertussis cases, with the highest risk in infants under two months old, before they can be vaccinated. Infants born to mothers vaccinated at least a week before delivery had a 91% lower risk of contracting pertussis [2]. Pertussis vaccination has been recommended for pregnant women in Great Britain since 2012, influenced by a 2012 outbreak [2]. In 2016, the recommendation was adjusted to vaccinate between the 20th and 32nd week of pregnancy, leading to increased vaccination coverage and thereby reducing the number of pertussis cases in prematurely born children while still protecting full-term infants. Vaccination rates increased by approximately 20% between 2013 and 2020, with coverage of pregnant women in England reaching 70% in 2020 [2,3]. Unfortunately, over the years following the COVID-19 pandemic, these statistics have deteriorated, reaching 58.9% of vaccinated women in March 2024 [57,58]. The reason for such a significant decline in vaccination coverage was not clear, and furthermore, this decline coincided with a large increase in the number of pertussis cases in 2023–2024. Potential causes include deepening inequalities in access to healthcare services and organizational barriers, reduced perception of the risk of pertussis after the lockdown period, and increased skepticism towards vaccinations after the pandemic.

In other highly developed countries, the number of women vaccinated against pertussis during pregnancy increased significantly following the introduction of appropriate recommendations. A high vaccination rate, as much as 95%, was reported in the Netherlands. In Australia, Canada, and Belgium, approximately 70% of women surveyed after the introduction of the recommendation were vaccinated against pertussis during pregnancy [16,31,59,60].

The recurring pertussis epidemics every few years have had a significant impact on the increase in vaccination coverage among pregnant women. On the one hand, they influenced the introduction of recommendations for pertussis vaccination in many countries. On the other hand, they independently increased interest in vaccinations among pregnant women. For example, in New Zealand, during the 2011–2014 epidemics, hundreds of infants were hospitalized due to pertussis, and three children died. This led to the introduction of vaccination recommendations, resulting in an increase in the number of vaccinated women by over 30% [42,61]. Following the introduction of maternal pertussis vaccination in Spain, vaccine uptake rapidly increased and remained high, reaching approximately 84% nationally in 2019 and around 85–90% in several regions in subsequent years [3,62,63].

Data from Italy show vaccination coverage ranging from 23% to 60%, depending on the study. Significantly more women were willing to be vaccinated if they received a recommendation from a doctor [64,65,66]. Unfortunately, many doctors still do not discuss vaccinations with pregnant women. This may be due to a lack of sufficient knowledge about vaccine safety or a lack of time to discuss vaccinations during prenatal visits. Studies in Korea confirm this, where nearly 30% of pregnant women did not receive any information about recommended vaccinations [41,67]. However, it is important to remember the limitations of these publications, primarily due to their regional nature, limited geographical representativeness, and lack of full control over confounding factors.

The availability of vaccinations is a key factor in coverage rates. Despite the benefits and the WHO’s recommendation for Tdap vaccination during pregnancy, most South and Southeast Asian countries still recommend the TT vaccine for pregnant women [68]. Since 2017, Tdap vaccination has been recommended in Singapore between 16 and 32 weeks of pregnancy [69]. Also in Vietnam, the Ministry of Health has approved the use of Tdap vaccines—Boostrix in 2022 and Adacel in 2024—for administration during pregnancy [70]. Although countries like India or Thailand have scientific societies or expert recommendations, low vaccination coverage persists due to limited healthcare access, lack of knowledge, and insufficient recommendations from healthcare providers [68,71,72].

Before the COVID-19 pandemic, global vaccination coverage had been steadily increasing over the past two decades, with the third dose of DTP reaching a vaccination rate of 86% by 2018. However, the pandemic saw significant disruptions to vaccination programs, with vaccination coverage falling to 83% in 2021. By 2023, global vaccination coverage with the third dose of DTP (DTP3) had slightly increased to 84%, still not reaching pre-pandemic levels in 2019 [57]. Population-level pertussis vaccination coverage provides important context for interpreting maternal vaccination uptake, as it reflects broader public attitudes toward immunization, vaccine confidence, and the effectiveness of national vaccination programs. Trends in maternal pertussis vaccination are often influenced by the same factors that affect vaccination coverage in the general population, including vaccine hesitancy, access to healthcare services, and public health policies. Therefore, population-level vaccination data may help explain observed patterns in vaccine acceptance among pregnant women. Interestingly, a modeling study by Zhang et al. found that global maternal pertussis vaccination coverage has shown an inverted U-shaped trend, increasing between 2015 and 2019 and decreasing between 2020 and 2023. This decline has been even more pronounced in high-income countries: the maternal pertussis vaccination rate in the US declined by almost 13% between 2019 and 2023 [73].

A systematic review by Patel et al. included 25 studies on increasing pertussis vaccination rates among pregnant women. The authors found multi-level interventions combining activities targeting patients, healthcare providers, and healthcare organizations to be the most effective. Interactive patient education, requiring active engagement, yielded better results than passive information delivery, and on-site vaccination increased accessibility and convenience, resulting in higher vaccination rates [74].

Numerous investigations have confirmed the favorable safety profile of prenatal Tdap vaccination, with no evidence of increased risk of adverse maternal, fetal, or neonatal outcomes [75,76].

### 3.3. Vaccination Against SARS-CoV-2

Contracting COVID-19 during pregnancy is dangerous for both the woman and the fetus. Due to the characteristic changes in anatomy, physiology, and the hormonal system that occur in a pregnant woman’s body, there is a higher risk of serious complications. These can include severe pneumonia, respiratory failure requiring intubation and intensive care, a higher incidence of preeclampsia or pulmonary embolism, which can increase the risk of death. COVID-19 may also have adverse consequences for the fetus, such as growth restriction, miscarriage, premature delivery with complications of prematurity, and even fetal death. A very severe course of illness has also been observed in young children during the first months of life [77,78,79].

Major medical societies in obstetrics, gynecology, and public health generally recommend COVID-19 vaccination during pregnancy. The CDC’s COVID-19 vaccination recommendations have been updated, and currently (2025–2026 recommendations) are based on an individual decision-making process that emphasizes weighing the benefits and risks of vaccination [80]. COVID-19 vaccination is most beneficial for individuals at increased risk of severe disease. The American College of Obstetricians and Gynecologists (ACOG) has recommended COVID-19 vaccination for pregnant women since January 2021. This position was upheld in March 2026 [81]. Vaccination is possible during any trimester of pregnancy, although the risk of severe COVID-19 is particularly high in the later stages of pregnancy, so it is advised to get vaccinated before reaching this period. Similar to the flu and pertussis vaccines, antibodies are produced after receiving the COVID-19 vaccine. These antibodies protect the pregnant woman against illness and severe infection, and they also pass through the placenta, protecting the fetus and newborn [77,78,79].

Significant differences in vaccine acceptance have been observed across countries [4,82,83]. The main reasons for low acceptance include fears of adverse effects of the vaccine, particularly referring to the unborn child [84]. Low awareness and a tendency to downplay the severity of the disease also contribute. Although COVID-19 vaccines were developed and brought to market in record time and subjected to rigorous clinical trials, false and negative opinions about vaccinations, often spread through the media and by family or friends, have significantly fueled concerns about vaccine safety and effectiveness [4,12,82,83,85]. Another important factor was the presence of misinformation among healthcare workers about COVID-19 vaccination during pregnancy [11]. Meanwhile, clear and positive communication about vaccinations by medical professionals had a significant positive impact on the decision to vaccinate [85,86].

During the COVID-19 pandemic, in some countries, there was a significant rise in interest in vaccinations, not only against SARS-CoV-2 but also those routinely recommended before the pandemic. The level of knowledge and awareness about vaccinations increased, often reducing fears and strengthening confidence in vaccines. In China, where high acceptance of COVID-19 vaccinations was demonstrated, there was an increasing willingness to receive the flu vaccine during pregnancy during the pandemic, suggesting an increase in awareness on this topic [87]. Studies conducted in Italy and the Netherlands during the pandemic found that educated women were more willing to be vaccinated during pregnancy [88,89]. On the other hand, influenza vaccination uptake among pregnant women remained relatively stable across pre-pandemic and COVID-19 periods in some settings, with rates around 50% reported in Israel before and during the pandemic. However, cohort studies indicate temporal variation in uptake rather than direct measurement of changes in individual attitudes [35,90].

Many studies draw attention to the connection between the acceptance of various vaccinations. A cross-sectional online questionnaire study conducted between September 2021 and May 2022 in the Netherlands found that women who were reluctant to get vaccinated against the flu were also hesitant about the COVID-19 vaccination. However, women who were vaccinated against influenza during pregnancy and/or against pertussis were more willing to accept vaccination against COVID-19 [89]. A retrospective cross-sectional study conducted in the US analyzed risk factors for declining antepartum vaccination and included three vaccinations during pregnancy: COVID-19, Tdap, and influenza. The acceptance rate was lowest for the COVID-19 vaccine at 46.1% compared to 58.0% for the influenza vaccine and 80.8% for the Tdap vaccine. Older age and nulliparity were independently associated with increased acceptance of COVID-19. Public insurance in pregnancy was associated with a decreased likelihood of vaccine acceptance for the COVID-19 and influenza vaccines. It is worth mentioning that this study is limited by its retrospective, single-center design, as well as information bias and lack of individual motivations underlying vaccination decisions, limiting interpretation to associations rather than underlying causes [91].

As with influenza and pertussis vaccinations during pregnancy, providing reliable information and recommending COVID-19 vaccination by healthcare workers, particularly doctors, had a significant impact. Higher education levels, higher socioeconomic status, older age, and information campaigns also played important roles [10,89,90]. It is important to provide patients with clear and transparent information about vaccinations, communicate without lecturing or judging them, and calmly address any doubts [92]. Rapidly changing clinical guidelines regarding COVID-19 vaccination in pregnant women may influence vaccination statistics, primarily through effects on healthcare provider recommendations and patient confidence. Inconsistencies or frequent updates in official guidance can increase uncertainty among both clinicians and pregnant individuals, potentially leading to delayed or deferred vaccination decisions. However, this effect is generally indirect and secondary to broader determinants such as overall vaccine acceptance, trust in healthcare systems, and epidemiological context.

Current evidence indicates that COVID-19 vaccination is not associated with an increased risk of adverse maternal, pregnancy, or infant outcomes [93].

### 3.4. Vaccination Against RSV

In August 2023, the U.S. Food and Drug Administration (FDA) approved the unadjuvanted RSVPreF vaccine for use in pregnant women between 32 and 36 weeks of gestation. However, according to the manufacturer’s registration, the vaccine is intended for women between 24 and 36 weeks of pregnancy. This difference reflects distinct regulatory risk-management approaches rather than conflicting assessments of vaccine efficacy or safety. Vaccination during pregnancy enhances the transplacental transfer of maternal RSV-specific antibodies, providing passive protection to infants against RSV infection during the first 3–6 months of life. At present, the unadjuvanted RSVPreF vaccine is the sole RSV vaccine indicated for maternal immunization. Among infants followed for 24 months after birth, rates of prematurity, low birth weight, developmental delay, and other serious adverse events were similar between those born to mothers who received the RSVpreF vaccine during pregnancy and those born to mothers who received a placebo. These findings support the long-term safety of maternal RSVpreF vaccination [94,95,96]. In a phase 3, randomized, double-blinded, placebo-controlled trial, vaccine efficacy, defined as protection against newborn and infant severe RSV-associated medically attended lower respiratory tract illness, was 82.4% and 70.0% within 90 and 180 days of birth, respectively [97].

Recommendations for RSV vaccination in subsequent pregnancies are currently a topic of debate. According to current CDC guidance, repeat maternal RSV vaccination is not recommended in subsequent pregnancies for women who have previously received an RSV vaccine during an earlier pregnancy [98]. Meanwhile, NHS recommendations state that vaccination should be given in all subsequent pregnancies [99].

Due to the recent introduction of maternal RSV vaccination, research on attitudes toward vaccination is limited. Results from a study in Gambia revealed a critically low level of mothers’ awareness of RSV: only 12.8% of participants reported being familiar with the virus, and only 10.6% recognized its potential to cause life-threatening respiratory disease in infants. Healthcare providers (82.6%) were the primary source of information. Paradoxically, willingness to receive the RSV vaccine during pregnancy was higher compared to the lactation period, primarily due to trust in clinical recommendations. The main barriers were safety concerns (88.8%), lack of vaccine availability (41.9%), and limited awareness (79.3%). Multiparity significantly correlated with acceptance of the pregnancy vaccine (*p* = 0.004), while higher education predisposed to RSV awareness (*p* = 0.022) [100]. However, this single-centre study has several limitations (e.g., cross-sectional design, possible selection bias, intention–behavior gap) that should be considered when interpreting the findings.

A study from the United States included pregnant women or those trying to conceive and explored their perceptions of RSV-related illnesses and their intention to get vaccinated against RSV. Logistic regression analysis was performed to determine the probability and predicted proportions of RSV vaccination during pregnancy, adjusting for sociodemographic factors. The study included over 15,000 online surveys. Fifty-four percent of respondents indicated they were “very likely” to get vaccinated against RSV during pregnancy. Perceiving RSV as a serious disease was the strongest predictor of their likelihood of getting vaccinated [101]. Similar to the study by Jasseh et al., this study is also cross-sectional and potentially addresses the intention–behavior gap.

A cross-sectional study conducted in the second half of 2024 in Italy examined the associations between socioeconomic factors and vaccination behavior. Education, employment status, and number of children were significantly associated with higher vaccination rates. For most women, the main source of information (60.5%) was the gynecologist. The principal barriers to vaccine uptake included insufficient discussion with healthcare professionals and concerns about vaccine safety. Despite these reservations, 83.8% of women expressed willingness to receive all recommended vaccines concomitantly, while 86.4% indicated a preference for maternal vaccination over the administration of monoclonal antibodies to their newborns [102].

In an Australian study including 440 pregnant women, attitudes toward a free RSV vaccine were evaluated. Latent class analysis revealed two distinct groups characterized as “acceptors/considerers” and “rejectors.” The acceptor/considerer group, comprising 68.3% of participants, was more likely to include older women, those with higher household incomes, and those who had received pertussis vaccination during pregnancy, whereas influenza vaccination was less common in this group. Notably, vaccine cost did not affect the preferences of women classified as rejectors. Similar to the observations of Lubrano et al., maternal RSV vaccination was preferred significantly more often than infant immunoprophylaxis with monoclonal antibodies (54.8% vs. 16.8%, respectively; *p* < 0.001) [103].

Even though vaccination of pregnant women against RSV is a relatively new recommendation from scientific and medical societies, based on the research conducted so far, we see that similar internal and external factors influence the decision to vaccinate. Because RSV vaccines for pregnant women were not yet widely available during the peak of the COVID-19 pandemic, it is impossible to assess the direct impact of the pandemic on vaccine acceptance and vaccination coverage. However, this impact may have been indirect, for example, through increased awareness of the risks associated with respiratory infections among pregnant women, newborns, and children.

It is also noteworthy that, according to current recommendations of the American Advisory Committee on Immunization Practices (ACIP), the RSV vaccine may be administered at any time before or after other vaccines routinely recommended for pregnant women (e.g., the pertussis vaccine). Most national advisory committees on immunization practices agree that these vaccines can, and sometimes even should, be administered during a single visit on the same day [104].

An overview of the major studies discussed in this article is provided in Table S1.

## 4. Discussion

Vaccination coverage among pregnant women is determined by clinical, behavioural, and health system-level factors. Healthcare provider recommendation, trust in medical institutions, vaccine availability, and maternal education are consistently identified as key predictors. However, these determinants exert heterogeneous effects depending on vaccine type, healthcare model, geographical diversity, and the phase of public health emergencies such as the COVID-19 pandemic. During the COVID-19 pandemic, there has been a noticeable increase in public awareness about vaccinations, but there have also been concerns regarding the introduction of new vaccines. In countries with official vaccination recommendations, where healthcare workers provided reliable information and encouraged vaccination, higher levels of acceptance of vaccinations during pregnancy were observed. Additionally, older individuals and those with chronic diseases were more willing to be vaccinated. People with higher education levels and socioeconomic status were also more open to vaccinations.

Variability between countries reflects differences in the organization of healthcare systems and prenatal care pathways. The degree of integration of preventive interventions into routine obstetric care is also important. Facilities with well-structured prenatal care programs, clear national vaccination schedules for pregnancy, and strong involvement of primary care providers demonstrate higher vaccination rates. Fragmented care models and inconsistent dissemination of guidelines, on the other hand, are associated with missed opportunities for vaccination during pregnancy, even when vaccine availability is adequate. These findings highlight that vaccination rates are not solely dependent on patients but are strongly influenced by clinical procedures at the systemic level. Much evidence demonstrates that professional organization of vaccination programs and systematic educational initiatives may be key to improving vaccination rates among pregnant women, not just a sudden shift in public beliefs. To improve vaccination rates among pregnant women, promotional activities targeting not only pregnant women but also medical staff are important. Doctors, midwives, and other healthcare workers should be aware of the latest guidelines on vaccinations during pregnancy and communicate this information to their patients in a clear, understandable way. They should address all patients’ doubts with empathy and in a non-judgmental manner. These approaches have proven effective in addressing concerns and promoting a more receptive attitude toward vaccination. Media campaigns that provide reliable information about the benefits of vaccinations and scientific research confirming their safety are also crucial and should be aimed at the general public. Taking into account the changing realities and the increasing importance of social media in shaping views and behaviors, it is worth considering this medium as a potential new source of information that should be verified and reliable. Disinformation, particularly amplified through digital platforms and social media, poses an increasingly significant clinical challenge impacting patient counseling and vaccine acceptance [105]. Exposure to information unsupported by scientific evidence can undermine trust in healthcare professionals and complicate shared decision-making processes in prenatal care. This reinforces the importance of organized, evidence-based communication strategies implemented by physicians, incorporating risk communication principles and addressing vaccination-related issues in a patient-centered manner. The importance of personalized medical advice should not be underestimated, as the attending physician is often the primary source of guidance and recommendations during pregnancy.

The COVID-19 pandemic has posed one of the greatest health challenges of the 21st century. It has also exposed the consequences of the emergence of a dangerous new pathogen and the disruption of the global healthcare system. It substantially altered prenatal care delivery and vaccination pathways. Limited clinician–patient interactions resulted in limited opportunities for vaccine counselling and administration. It should also not be overlooked that changes in risk perception related to the pandemic have influenced mothers’ decision-making. During ongoing and future pandemics, health services should prioritize equitable access to healthcare, including routine immunizations.

Global data show that health inequalities remain a persistent factor in differential vaccination rates in pregnancy. Limited access to organized prenatal care, delayed prenatal appointments, socioeconomic disadvantage, and limited health literacy are associated with lower vaccination rates. Furthermore, interruptions in the continuity of care further reduce the likelihood of receiving counseling and vaccine administration. Providing easy access to vaccinations is as important as education.

From an implementation perspective, improving vaccination coverage among pregnant women requires integrating vaccination services into routine prenatal care routines. This includes, among other things, ensuring clinician training on current vaccination guidelines. Counseling by midwives and obstetricians remains a key intervention, especially when conducted consistently and in accordance with national clinical guidelines.

At the systemic level, reducing logistical and financial barriers—such as the cost of vaccines, distance to healthcare facilities, and limited availability of appointments—remains crucial to improving equity.

In most countries, there are no official statistics on vaccination coverage among pregnant women for specific pathogens. Regular monitoring of the number of vaccinated pregnant women and the outcomes of these vaccinations is important for assessing the effectiveness of implemented programs and adapting appropriate vaccination strategies. National registries of vaccinations among pregnant women would provide future researchers with precise data on vaccination coverage in specific regions of the world and the effectiveness of actions aimed at increasing vaccination uptake. It is also important to determine what specific factors influenced the disruption of the vaccination process during and after the pandemic in order to be able to draw conclusions for the future.

The determinants identified in this review can also be interpreted within the framework of vaccine hesitancy models proposed by the World Health Organization, particularly the 3Cs model (Confidence, Complacency, Convenience) and its expanded 5Cs version (Confidence, Complacency, Constraints, Calculation, and Collective Responsibility) [106]. Confidence refers to trust in vaccine safety and effectiveness, healthcare professionals, and public health institutions. In our review, healthcare provider recommendations, trust in medical institutions, and exposure to reliable information emerged as major factors influencing vaccination uptake during pregnancy. Complacency reflects the perceived risk of vaccine-preventable diseases. During the COVID-19 pandemic, changing perceptions of infection risk influenced maternal decision-making, with higher perceived susceptibility often associated with greater willingness to vaccinate in some regions. Convenience, or Constraints in the 5Cs model, encompasses practical barriers such as vaccine availability, accessibility of healthcare services, financial costs, and continuity of prenatal care. Consistent with this framework, logistical obstacles, fragmented healthcare pathways, and delayed prenatal visits were associated with lower vaccination rates. The Calculation component describes individuals’ tendency to seek extensive information and weigh perceived risks and benefits before making vaccination decisions. This dimension became particularly relevant during the COVID-19 pandemic, when pregnant women were exposed to large volumes of both scientific information and misinformation through digital media. Finally, Collective Responsibility refers to the willingness to protect others through vaccination. Pregnancy vaccination decisions are often primarily motivated by concerns for maternal and infant health. Applying the 3Cs/5Cs framework helps to contextualize vaccine hesitancy among pregnant women as a multifactorial phenomenon shaped by individual perceptions, healthcare-system characteristics, and social influences, highlighting the need for multidimensional interventions to improve vaccination coverage.

Main limitations of this narrative review include heterogeneity in study designs and outcomes, reliance on self-reported vaccination data, variability in evolving clinical guidelines across time, and methodological constraints related to database selection and language restrictions.

## 5. Conclusions

To improve vaccination rates among pregnant women, it is essential to provide clear, evidence-based information through healthcare professionals, particularly those directly caring for pregnant women. Educational campaigns should address concerns calmly and without judgment, emphasizing the safety and benefits of vaccinations. The role of social media in promoting health, including vaccinations for pregnant women, should not be overstated. Healthcare professionals’ recommendations remain the most effective communication strategy regarding vaccinations in the pre- and post-COVID era. Enhanced access to healthcare and vaccinations, along with strategic information dissemination, can significantly improve vaccine acceptance during pregnancy. Lessons learned from past pandemics should be incorporated into the development of healthcare strategies aimed at implementing recommended vaccinations for pregnant women in the future.

## Data Availability

No new data were created or analyzed in this study. Data sharing is not applicable to this article.

## Supplementary Materials

The following supporting information can be downloaded at https://www.mdpi.com/article/10.3390/vaccines14060536/s1. Table S1: Overview of the most relevant research discussed in this article.

## Abbreviations

The following abbreviations are used in this manuscript:

3Cs	Confidence, Complacency, Convenience
5Cs	Confidence, Complacency, Constraints, Calculation, and Collective Responsibility
ACIP	Advisory Committee on Immunization Practices
CDC	Centers for Disease Control and Prevention
CI	Confidence Interval
DTP	Diphtheria, Tetanus, Pertussis vaccine
ECDC	European Centre for Disease Prevention and Control
FDA	Food and Drug Administration
IgG	Immunoglobulin G
MeSH	Medical Subject Headings
NHS	National Health Service
RSV	Respiratory syncytial virus
RSV-LRTD	RSV-induced lower respiratory tract disease
TT	Tetanus Toxoid
WHO	World Health Organization
