# Supplementary material for: Maternal Vaccine Acceptance and Attitudes Before and After the COVID-19 Pandemic: A Narrative Literature Review
Source: Vaccines (Basel). 2026 Jun 17;14(6):536. doi: 10.3390/vaccines14060536 (PMC13307573; doi:10.3390/vaccines14060536)
Supplement: Supplementary file 1 [file vaccines-14-00536-s001.zip › vaccines-4336499-supplementary.pdf]

Table S1. Overview of the most relevant research discussed in this article.

Type of Vaccine	Study/Population/Country/Duration	Beneficial Effect	Negative Effect
Influenza vaccine	King JP et al. (2020), pregnant women, USA, 2016–2017	Positive beliefs and attitudes towards vaccines correlated with higher uptake	-
	Carlson S et al. (2020), pregnant women, Australia, 2016	Effective public health campaigns increased awareness and uptake	-
	Baïssas T et al. (2021), pregnant women, UK, USA, Spain	High-performing vaccine programs in these countries demonstrated improved vaccination uptake	-
	Pisula A et al. (2022), pregnant women, Poland	Positive attitudes towards vaccination linked to awareness campaigns	-
	Hong K et al. (2022), pregnant women, USA, 2010-2018	Private insurance coverage correlated with higher vaccination rates	-
	Jiang F et al. (2022), pregnant women, China, 2022	Knowledge about pertussis vaccination was positively associated with the acceptance of influenza vaccination	-
Pertussis vaccine	Otieno NA et al. (2020), pregnant women, Kenya, 2019-2020	Educational campaigns and awareness increased vaccine acceptance	-

	Howe AS et al. (2020), pregnant women, New Zealand, 2020	Combining education and vaccination services enhanced coverage	-
	Kim C et al. (2021), pregnant women, Korea, 2021	Government recommendations improved vaccine uptake	-
	Vilca LM et al. (2021), pregnant women, Italy, 2021	Differences in education and awareness between regions impacted vaccination rates	-
SARS-CoV-2 vaccine	Tao L et al. (2021), pregnant women, China	High acceptance based on health belief model	-
	Husain F et al. (2022), pregnant women, UK	High vaccine uptake among socially and ethnically diverse pregnant women	-
	Hui L et al. (2023), pregnant women, multi-country study	Reduction in stillbirths and preterm births among vaccinated women	-
	De Brabandere L et al. (2023), pregnant and lactating women, global review	Social media and pandemic increased awareness towards vaccinations	Hesitancy influenced by misinformation
	Alderotti G et al. (2023), vaccinators, Italy	Effective communication strategies identified for discussing COVID-19 vaccination with patients	-
